# Ibudilast Attenuates Folic Acid–Induced Acute Kidney Injury by Blocking Pyroptosis Through TLR4-Mediated NF-κB and MAPK Signaling Pathways

**DOI:** 10.3389/fphar.2021.650283

**Published:** 2021-05-07

**Authors:** Xue Li, Yu Zou, Yuan-Yuan Fu, Jia Xing, Kai-Yue Wang, Peng-Zhi Wan, Mo Wang, Xiao-Yue Zhai

**Affiliations:** ^1^ Department of Histology and Embryology, Basic Medical College, China Medical University, Shenyang, China; ^2^ Department of Nephrology, Shengjing Hospital of China Medical University, Shenyang, China; ^3^ Department of Nephrology, First Affiliated Hospital of China Medical University, Shenyang, China; ^4^ Department of Surgery, Yale School of Medicine, New Haven, CT, United States; ^5^ Institute of Nephropathology, China Medical University, Shenyang, China

**Keywords:** ibudilast, folic acid–induced acute kidney injury, pyroptosis, inflammation, toll-like receptor 4, NF-κB, mitogen-activated protein kinase

## Abstract

Folic acid (FA)-induced renal tubule damage, which is characterized by extensive inflammation, is a common model of acute kidney injury (AKI). Pyroptosis, a pro-inflammatory form of cell death due to the activation of inflammatory caspases, is involved in AKI progression. Ibudilast, a TLR4 antagonist, has been used in the clinic to exert an anti-inflammatory effect on asthma. However, researchers have not explored whether ibudilast exerts a protective effect on AKI by inhibiting inflammation. In the present study, ibudilast reversed FA-induced AKI in mice, as indicated by the reduced serum creatinine and urea nitrogen levels, and improved renal pathology, as well as the downregulation of kidney injury marker-1. In addition, ibudilast significantly increased the production of the anti-inflammatory factor IL-10 while suppressing the secretion of the pro-inflammatory cytokine TNF-α and macrophage infiltration. Moreover, in the injured kidney, ibudilast reduced the levels of both inflammasome markers (NLRP3) and pyroptosis-related proteins (caspase-1, IL1-β, IL-18, and GSDMD cleavage), and decreased the number of TUNEL-positive cells. Further mechanistic studies showed that ibudilast administration inhibited the FA-induced upregulation of TLR4, blocked NF-κB nuclear translocation, and reduced the phosphorylation of NF-κB and IκBα, p38, ERK, and JNK. Thus, this study substantiates the protective effect of ibudilast on FA-induced AKI in mice and suggests that protection might be achieved by reducing pyroptosis and inflammation, likely through the inhibition of TLR4-mediated NF-κB and MAPK signaling pathways.

## Introduction

Acute kidney injury (AKI) is a severe clinical syndrome that rapidly causes renal dysfunction and contributes to a high mortality rate (Bellomo et al., 2012). Approximately 13.3 million people are affected worldwide, and approximately 2 million die of AKI each year (Mehta et al., 2015). Renal tubules are vulnerable to various injuries, including hypoxia, drugs, and toxins (Linkermann et al., 2014). FA-induced tubular injury is a well-known experimental model for studying AKI. The central pathological features underlying FA-induced AKI are tubular obstruction and oxidative stress, which trigger tubular epithelial cell (TEC) necrosis and cytokine release (Aparicio-Trejo et al., 2019).

Pyroptosis, a pro-inflammatory form of programmed cell death, results in the release of large amounts of inflammatory cytokines (Samir et al., 2019). Pyroptosis is induced by various damage-associated molecular patterns (DAMPs) or pathogen-associated molecular patterns (PAMPs), which trigger inflammation by binding pattern recognition receptors (Rashidi et al., 2019). Notably, the pyrin domain–containing 3 (NLRP3) inflammasome, one member of the NOD-like receptor family, is an essential cytosolic pattern recognition receptor associated with inflammation in various kidney diseases (Hutton et al., 2016; Li C. et al., 2019; Wang Y. C. et al., 2019). Once the NLRP3 inflammasome is activated, the N-terminal pyrin domain (PYD) of the NLRP3 protein containing a caspase recruitment domain acts as the platform for caspase-1 activation (Kim et al., 2019). Activated caspase-1 mediates the proteolytic cleavage of the pro-forms of interleukin 1β (pro-IL-1β) and IL-18 (pro-IL-18) into the active forms (Tajima et al., 2019). In addition, active caspase-1 cleaves the full-length gasdermin D (GSDMD-FL) protein to the pore-forming domain (GSDMD-N275), resulting in the insertion of the N-terminal domain into cell membranes and subsequent assembly into large oligomeric structures to permeabilize the membrane (Ma et al., 2019). GSDMD is thus an executioner mediating cell lysis and the release of mediators of inflammation (Mulvihill et al., 2018). Unlike noninflammatory apoptosis, pyroptosis is characterized by caspase-1 dependence, the formation of pores in the plasma membrane, cell swelling, and cytolysis, leading to DNA fragmentation and the release of inflammatory factors (Wu et al., 2016). Thus, it is a highly immunogenic process. Many studies have reported that pyroptosis of tubular epithelial cells contributes to tubular necrosis and inflammation, which enhance kidney damage (Chung et al., 2012; Krautwald and Linkermann 2014; Lorenz et al., 2014).

Toll-like receptor 4 (TLR4), an important member of the TLR family, is a pattern recognition receptor expressed on the cell surface that not only recognizes exogenous microbes but also responds to endogenous factors, exerting an essential effect on the induction of pro-inflammatory responses (Wang Y. et al., 2019). Moreover, TLR4 typically signals mainly through its downstream nuclear factor kappa B (NF-κB) and mitogen-activated protein kinase (MAPK) signaling pathways (Ma et al., 2019; Alban et al., 2020). NF-κB, a nuclear transcription factor, is activated when it is translocated into the nucleus (Kim et al., 2019). Nuclear translocation is predominantly prevented by binding to the inhibitor of kappa Bα (IκBα), an inhibitor of NF-κB, while pathways targeting IκBα phosphorylation for rapid degradation promote the nuclear translocation of NF-κB (Al-Harbi et al., 2016). TLR4/NF-κB signaling is closely associated with immune and inflammatory alterations, and its inhibition may ameliorate neuroimmune disorders by decreasing the levels of pro-inflammatory mediators (Ahmad et al., 2017). Additionally, the suppression of NF-κB function exerts an anti-inflammatory effect on LPS-induced acute lung injury through the upregulation of anti-inflammatory cytokines and downregulation of pro-inflammatory factors (Ahmad et al., 2018). Emerging data suggest that the activation of TLR4/NF-κB signaling is involved in renal inflammation in ischemia-reperfusion (I/R)- or LPS-induced AKI (Sanz et al., 2010; Luo et al., 2017). Moreover, activation of the TLR/NF-κB signaling pathway has been shown to increase the expression of the NLRP3 inflammasome, caspase-1, and pro-inflammatory factors to induce pyroptosis (Luo et al., 2017; Chen et al., 2019; Li Z. et al., 2019). In addition, the TLR4-mediated MAPK cascade plays a key role in renal inflammation, and pharmacological agents targeting the MAPK pathway have been reported to serve as a therapy to reduce renal damage (Zhang et al., 2008). The MAPK signaling pathway consists of p38, c-Jun NH_2_-terminal protein kinases (JNK), and extracellular signal-related protein kinases (ERK), and the activated forms of these kinases participate in the activation of NLRP3 inflammasome, which further aggravates the inflammatory response (Li et al., 2018).

Ibudilast, a nonselective phosphodiesterase 4 (PDE4) inhibitor and TLR4 antagonist, is clinically used to treat asthma (Kawasaki et al., 1992). Recently, ibudilast was reported to exert a protective effect on neuroinflammation, such as in Parkinson’s disease, poststroke dizziness, and delirium, by downregulating TLR4 (Rolan et al., 2009; Zhaleh et al., 2014; Wieseler et al., 2017; Jalleh et al., 2012). Additionally, ibudilast alleviates the microglial cell-mediated inflammatory response caused by human immunodeficiency virus-1 by blocking the TLR4/NF-κB pathway (Kiebala and Maggirwar 2011). Moreover, ibudilast has been used as an inhibitor of macrophage migration inhibitory factor, which further suppresses MAPK signaling and inflammation, as a potential strategy for tumor immunotherapy in patients with glioblastoma (Alban et al., 2020). However, the effect of ibudilast on AKI remains largely undefined.

In this study, we determined the effect of ibudilast on FA-induced AKI in mice by mainly examining its anti-pyroptotic role, that is, anti-inflammatory scavenging in TLR4-mediated NF-κB and MAPK signaling pathways.

## Materials and Methods

### Reagents and Antibodies

Ibudilast and FA were purchased from Meilun Biotechnology Co. (Dalian, Liaoning, China). We used the following antibodies to assess protein expression: anti–KIM-1, anti-NLRP3, anti–caspase-1, anti-GSDMD, and anti–β-actin antibodies from Abcam (Cambridge, MA, United States of America); anti–p-p65, anti-p65, anti–*p*-IκBα, anti-IκBα, anti–*p*-ERK, anti-ERK, anti–p-p38, anti-p38, anti–*p*-JNK, anti-JNK, anti-F4/80, and anti–IL-1β antibodies from Cell Signaling (Danvers, MA, United States of America); and anti–IL-18, anti–TNF-α, and anti-TLR4 antibodies from Proteintech (Wuhan, China).

### Animals

All animals involved in the experiments were used according to the NIH Criteria for the Use of Laboratory Animals. This study was approved by the Ethics Committee of the China Medical University Institutional Animal Care and Use Committee (protocol no. 2011037). C57BL/6 mice (male, 6–8 weeks) were purchased from China Medical University (Liaoning, China). They were maintained at a controlled temperature and humidity on a 12 h light/dark cycle. Mice were randomly divided into 4 groups (*n* = 5 mice per group): 1) control group: vehicle was administered by intraperitoneal injection, 2) ibudilast group: ibudilast [10 mg/kg, dissolved in 35% poly (ethylene glycol) 400] was administered b.i.d. by intraperitoneal injection, 3) FA group: FA (250 mg/kg, dissolved in 300 mM sodium bicarbonate buffer) was administered by intraperitoneal injection once, and 4) FA + ibudilast group: ibudilast was administered 2 h prior to the FA injection for two consecutive days. Two days after the FA injection, kidney specimens and blood samples were collected.

### Assays of Renal Function

Serum creatinine and BUN levels were determined to evaluate renal function in accordance with the manufacturer’s protocols (Jiancheng, Nanjing, China).

### Enzyme-Linked Immunosorbent Assay

The serum samples and kidney tissue homogenate were analyzed according to the instructions of the ELISA kits (RayBiotech, Inc. Norcross, GA, United States) to detect the levels of IL-10.

### Renal Histopathology

The renal specimens were fixed with 4% paraformaldehyde for 24 h and embedded in paraffin. Sections were cut at a thickness of 3 μm, and then, histopathology was assessed by performing hematoxylin and eosin (HE) and periodic acid-Schiff (PAS) staining. For the quantification of morphological changes, we randomly selected at least 10 fields in HE-stained sections from each sample to evaluate tubular damage, such as tubular dilation, vacuolization, loss of the brush border, epithelial necrosis, and interstitial edema. The sections were scored as follows: no injury (0); <20% (1); 20–50% (2); 50–70% (3); and >70% injury (4) (Brooks et al., 2009).

### TUNEL Assay

For the TUNEL assay (Roche), renal slices were stained with an *In Situ* Cell Death Detection Kit according to the manufacturer’s protocols. The samples were cleared in xylene and rehydrated in ethanol. Then, antigen retrieval was performed, and endogenous peroxidase activity was reduced by incubation with 3% H_2_O_2_ in methyl alcohol. The slices were blocked with 5% BSA to reduce nonspecific binding and incubated with the TUNEL reaction mixture for 60 min. Finally, sections were counterstained with hematoxylin.

### Immunohistochemical Staining

After de-waxing and rehydration, antigen retrieval was performed at a high power, and sections were washed with PBS. Then, 3% H_2_O_2_ was used to inhibit endogenous peroxidase activity for 10 min, and sections were blocked with 5% BSA for 30 min. Immunohistochemistry was performed with the following antibodies at 4°C overnight: anti–KIM-1 (1:200), anti-NLRP3 (1:250), anti–caspase-1 (1:400), anti–IL-1β (1:150), anti–TNF-α (1:150), and anti-F4/80 (1:200). The slices were incubated with the secondary antibody for 60 min, stained with diaminobenzidine, and counterstained with hematoxylin.

### Immunofluorescence Staining

After deparaffinization, the slices were subjected to antigen retrieval, permeabilized with 0.3% Triton-X-100 for 10 min, blocked with 5% BSA for 30 min, and then probed with the following antibodies: anti–IL-18 antibody (1:100), anti-TLR4 antibody (1:150), anti-GSDMD antibody (1:200), and anti-p65 antibody (1:200). On the next day, the samples were incubated with FITC-conjugated or TRITC-conjugated secondary antibodies. Finally, DAPI was used to stain the nuclei for 5 min.

### Western Blot

Proteins were extracted from kidney tissues, and protein concentrations were measured. Equal amounts of protein samples were separated on SDS-PAGE gels and transferred to PVDF membranes; the membranes were then blocked with 5% BSA for 60 min at room temperature. The membranes were probed with the following primary antibodies at 4°C overnight: anti–KIM-1 (1:500), anti-NLRP3 (1:500), anti–caspase-1 (1:1000), anti-GSDMD (1:1000), anti–IL-1β (1:1000), anti–TNF-α (1:1000), anti-TLR4 (1:500), anti–p-p65 (1:1000), anti-p65 (1:1000), anti–*p*-IκBα (1:1000), anti-IκBα (1:1000), anti–p-p38 (1:1000), anti-p38 (1:1000), anti–*p*-ERK (1:1000), anti-ERK (1:1000), anti–*p*-JNK (1:1000), anti-JNK (1:1000), and anti–β-actin (1:3000). Then, the membranes were incubated with peroxidase-conjugated secondary antibodies at room temperature for 60 min and visualized using enhanced chemiluminescence (ECL) reagents. The densitometry results were normalized to the control β-actin.

### Real-Time Polymerase Chain Reaction

The kidney tissue (50 mg) was cut into pieces in 1 ml of TRIzol solution (Vazyme, Nanjing, China) and then incubated with 0.2 ml of chloroform on ice for 5 min. The supernatants were extracted *via* centrifugation at 12,000 rpm for 15 min and mixed with an equal volume of isopropanol on ice for 15 min. The mixtures were centrifuged at 12,000 rpm for 10 min, and then, the upper phase was discarded. Next, 1 ml of 75% ethanol/ml TRIzol was added, and the solutions were centrifuged at 8,000 rpm for 5 min. DEPC-treated water was added to resuspend the RNA. The RNA concentrations were determined and standardized to 1,000 ng/μl. Total RNA was reverse-transcribed (RT) to cDNAs using a PrimeScript RT reagent Kit (Vazyme). Subsequently, polymerase chain reaction (PCR) was performed with the resulting cDNAs and SYBR Green Mix (Vazyme) using a Roche 4800 RT-PCR detection system. The specific primers used in this study were as follows:

NLRP3 forward: 5′-ATG​CTG​CTT​CGA​CAT​CTC​CT-3′ and reverse:
5′-AAC​CAA​TGC​GAG​ATC​CTG​AC-3′; caspase1 forward:
5′-GAC​TGG​GAC​CCT​CAA​GTT​TT-3′ and reverse:
5′-CCA​GCA​GCA​ACT​TCA​TTT​CT-3′; IL-1β forward:
5′-CCC​TGC​AGC​TGG​AGA​GTG​TGG​A-3′ and reverse:
5′-CTG​AGC​GAC​CTG​TCT​TGG​CCG-3′; IL-18 forward:
5’-GAC​TCT​TGC​GTC​AAC​TTC​AAG​G-3′; and reverse:
5′-CAG​GCT​GTC​TTT​TGT​CAA​CGA-3’; TNF-α forward:
5′-GCG​GAG​TCC​GGG​CAG​GTC​TA-3′; and reverse:
5′-GGG​GGC​TGG​CTC​TGT​GAG​GA-3′; TLR4 forward:
5′-CCA​TGC​ATT​TGG​CCT​TAG​CC-3′ and reverse:
5′-TGC​AGC​AGT​CTA​CTG​TGT​GG-3′; and *β*-actin forward:
5′-GGC​TGT​ATT​CCC​CTC​CAT​CG-3′ and reverse:
5′-CCA​GTT​GGT​AAT​GCC​ATG​T-3′. The relative mRNA expression levels were determined using the 2^−ΔΔCt^ method.

### Statistical Analysis

All values were presented as the mean ± standard errors and were analyzed using SPSS software (version 21.0, Chicago, IL, United States). Differences between groups were evaluated using one-way ANOVA with the Bonferroni test. A *p*-value <0.05 was considered statistically significant.

## Results

### The Effects of Ibudilast on Kidney Function and Morphological Changes in Mice With Folic Acid–Induced Acute Kidney Injury

**FIGURE 1:**
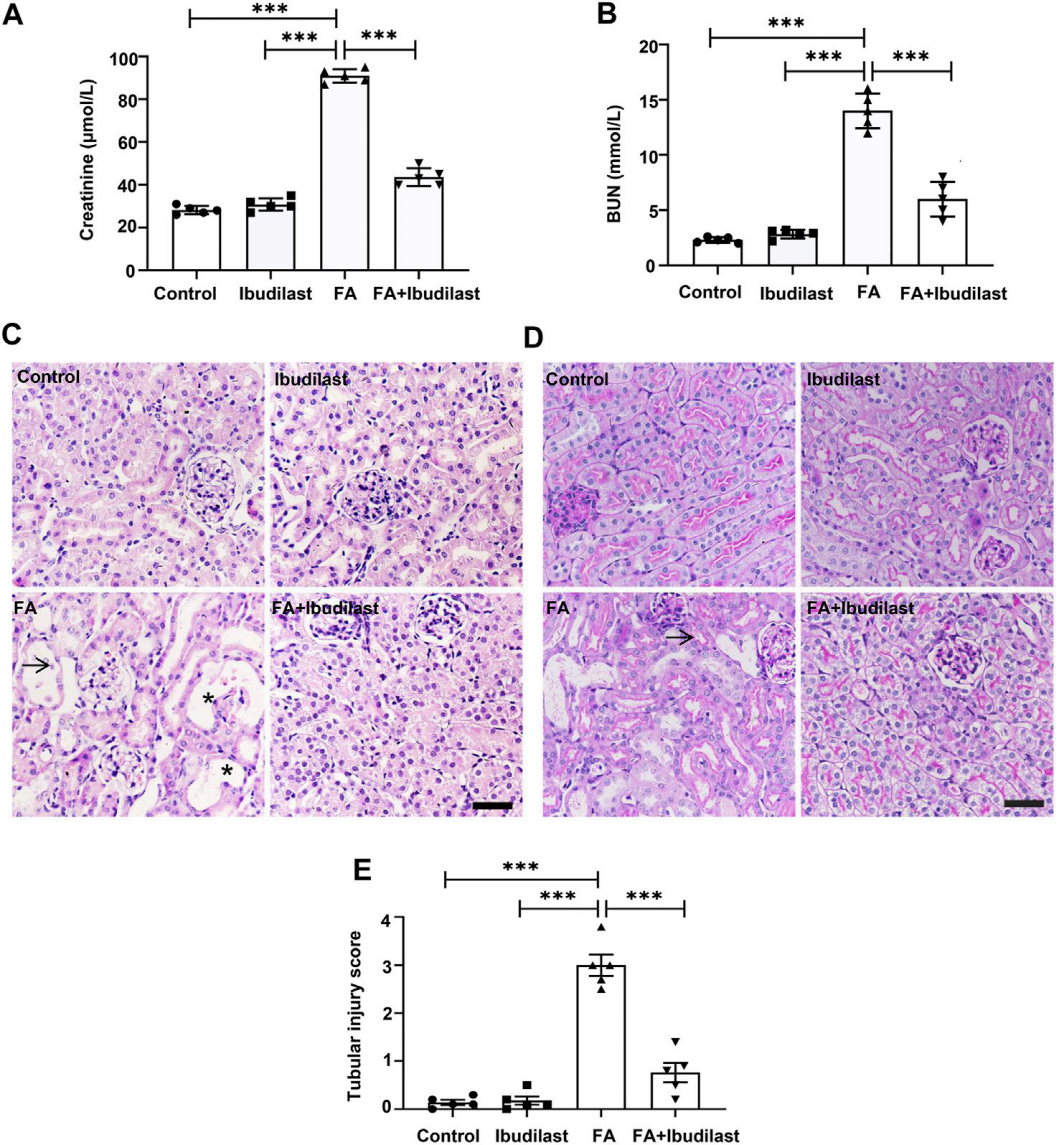
Ibudilast administration prevented FA-induced AKI. **(A)** Renal function was evaluated by measuring serum creatinine and **(B)** serum BUN levels. **(C)** Kidney tissue slices stained with HE, bar = 50 μm. Arrows indicate necrotic cells or cell debris; asterisks indicate dilated tubules. **(D)** Kidney tissue slices stained with PAS. The arrow indicates the brush border. **(E)** Tubular injury scoring was based on HE staining. Data are shown as the mean ± SEM, *n* = 5. *p < 0.05, **p < 0.01, and ***p < 0.001.

Renal function was measured to investigate the protective effect of ibudilast on FA-induced AKI, and the FA injection induced dramatic increase in serum creatinine and BUN levels to 91.1 ± 3.16 μmol/L and 13.9 ± 0.70 mmol/L, respectively, indicating impaired renal function (Figures 1A,B). These functional parameters associated with AKI were reduced to 43.6 ± 4.16 μmol/L and 6.0 ± 0.72 mmol/L following ibudilast administration, indicating the amelioration of renal function. Consistent with the decreased renal function, histological analyses with HE and PAS staining revealed acute tubular damage, including tubular dilatation, vacuolization, loss of the brush border, and shedding of epithelial cells in tubules in FA-injected mice, while ibudilast administration alleviated these pathological alterations, as shown in Figures 1C,D. Moreover, tubular injuries were assessed by quantification of HE-stained sections and revealed significantly increased tubulointerstitial injury scores (3.0 ± 0.22) in mice injected with FA injection, which were reduced by ibudilast administration (0.8 ± 0.20; Figure 1E).

### The Effects of Ibudilast on Tubular Injury in Mice With Folic Acid–Induced Acute Kidney Injury

**FIGURE 2:**
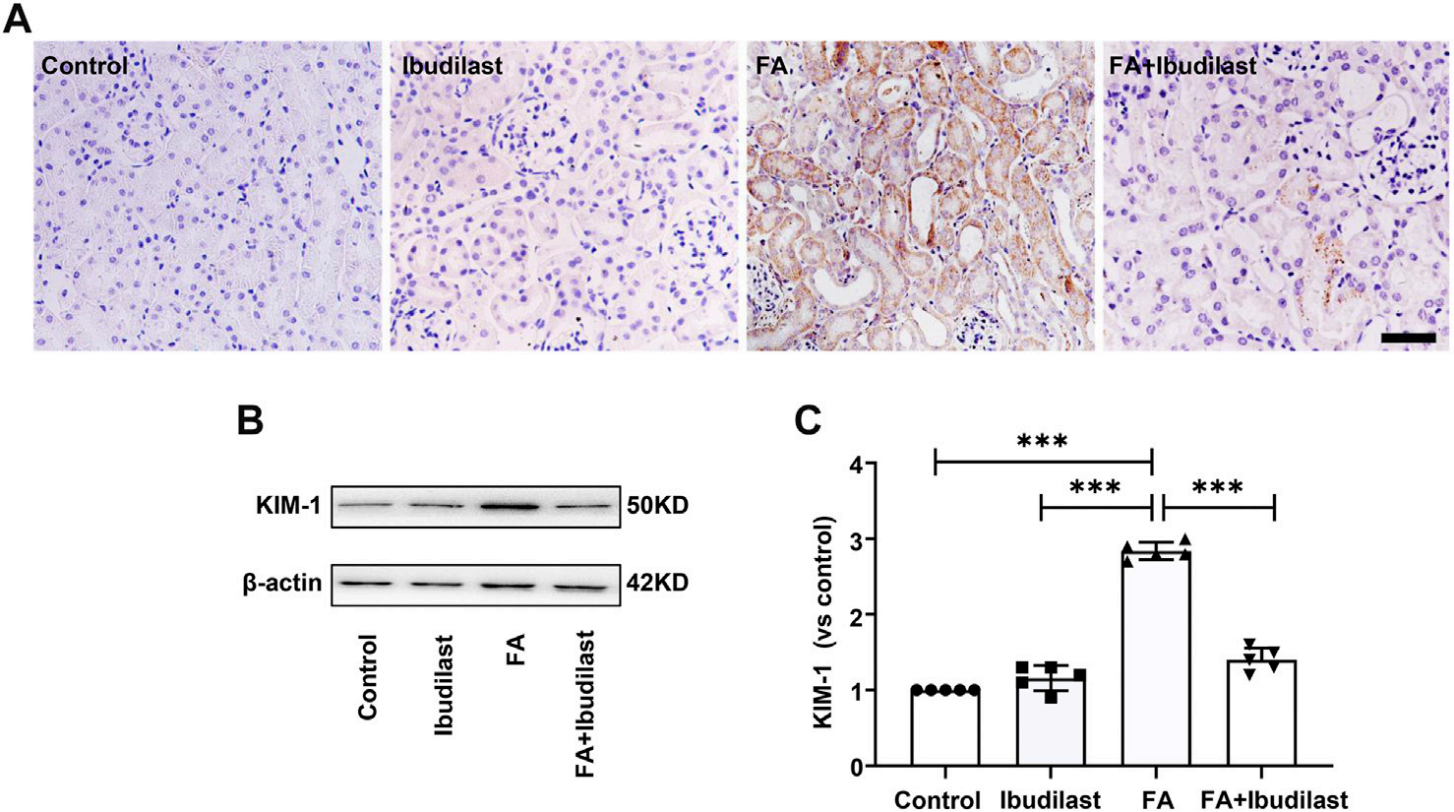
Ibudilast administration decreased the levels of the tubular injury marker KIM-1 in mice with FA-induced AKI. **(A)** Immunohistochemical labeling for KIM-1, bar = 50 μm. **(B)** Western blot for KIM-1. **(C)** Semiquantitative measurements of KIM-1. Data are presented as the mean ± SEM, *n* = 5. *p < 0.05, **p < 0.01, and ***p < 0.001.

Immunohistochemical staining was used to detect the tubular injury marker KIM-1 and to further assess tubular damage. FA-injected mice displayed significantly increased expression of KIM-1, which was mostly distributed in tubular epithelial cells, while ibudilast inhibited the expression of KIM-1 (Figure 2A). Similarly, Western blot analysis revealed that KIM-1 was present at a very low level in the control and ibudilast groups. Following FA injection, the level of KIM-1 was upregulated by approximately 2.8-fold, a change that was attenuated by 50.7% upon ibudilast administration (Figures 2B,C).

### Ibudilast Inhibited NLRP3 Inflammasome Activation and Reduced Pyroptosis in Mice With Folic Acid–Induced Acute Kidney Injury

**FIGURE 3:**
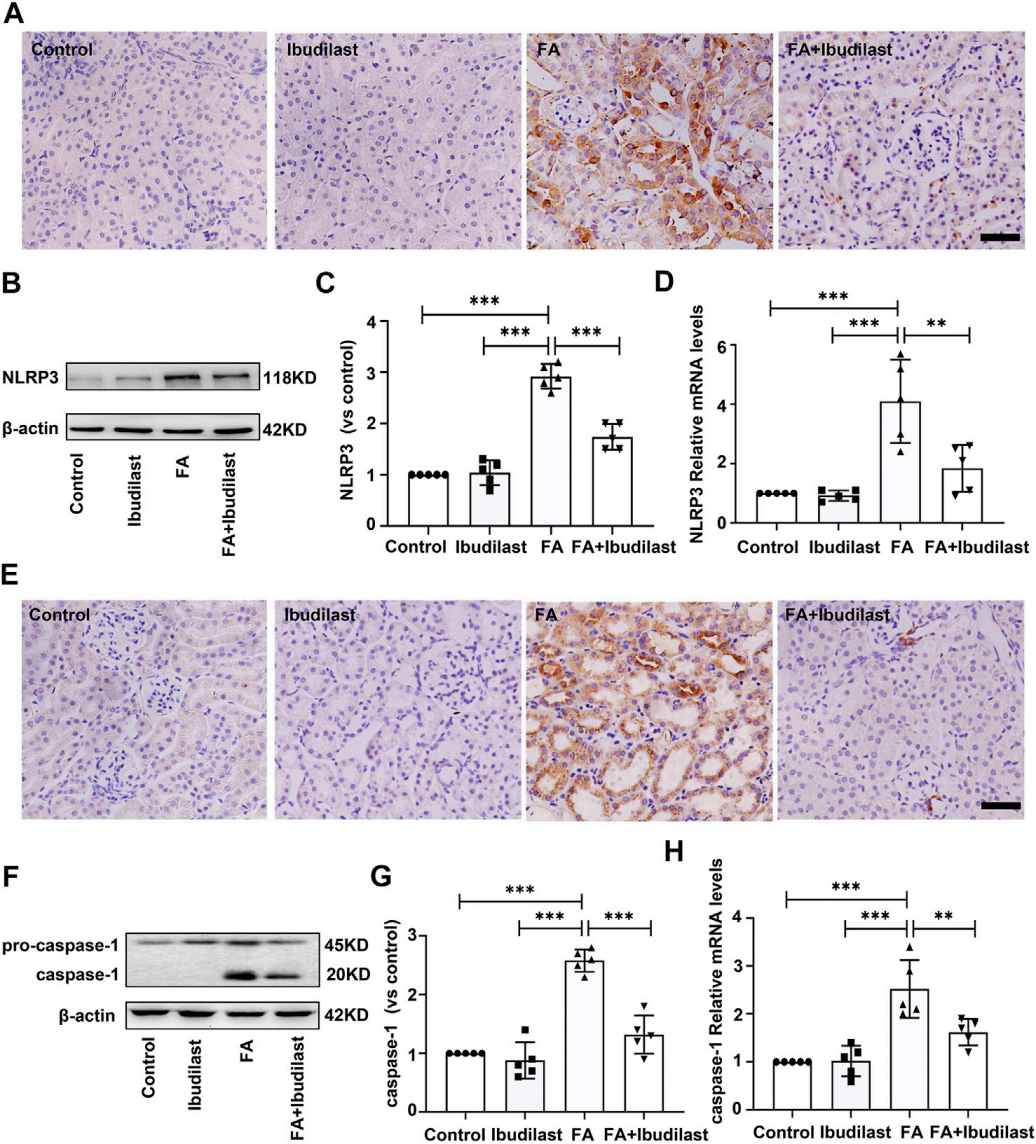
Ibudilast administration reduced NLRP3 inflammasome formation and caspase-1 activation in mice with FA-induced AKI. **(A)** Immunohistochemical labeling for the inflammasome marker NLRP3, bar = 50 μm. **(B)** Western blot for NLRP3. **(C)** Semiquantitative measurements of NLRP3. **(D)** Relative mRNA expression of NLRP3. **(E)** Immunohistochemical labeling of caspase-1, bar = 50 μm. **(F)** Western blot showing the levels of pro–caspase-1 and caspase-1. **(G)** Semiquantitative measurements of caspase-1. **(H)** Relative mRNA expression of caspase-1. Data are shown as the mean ± SEM, *n* = 5. *p < 0.05, **p < 0.01, and ***p < 0.001.

We measured the expression of NLRP3 using IHC staining to evaluate whether NLRP3 inflammasome activation was involved in FA-induced kidney injury and observed the upregulation of NLRP3 in FA-injected mice, which was predominantly located in the cytoplasm of tubular epithelial cells (Figure 3A). However, the expression of NLRP3 in ibudilast-treated mice was lower than that in FA-injected mice. Western blot analysis also supported this result, where an approximately 2.9-fold increase in NLRP3 expression was observed after FA injection, but ibudilast administration reduced the increase by 40.4% (Figures 3B,C). These changes were further confirmed through a gene expression analysis using RT-PCR (Figure 3D). Furthermore, we aimed to understand whether pyroptosis was initiated by inflammasome activation in FA-injured kidneys. We measured the level of the pyroptosis marker caspase-1 with IHC staining. Consistent with the expression and distribution of NLRP3, caspase-1 was activated in tubular epithelial cells, and its levels were elevated following FA injection. In comparison, the numbers of positive cells were decreased upon ibudilast administration (Figure 3E). This result was consistent with the Western blot findings, revealing that the FA injection increased the expression of caspase-1 by approximately 2.6-fold, while the level of pro–caspase-1 was not significantly affected. Moreover, we observed that ibudilast administration decreased the level of caspase-1 by approximately 48.9% and significantly suppressed its activation (Figures 3F,G). At the same time, FA injection increased the expression of caspase-1 mRNA by approximately 2.2-fold compared with basal levels: a change that was reduced by 35.7% after ibudilast administration (Figure 3H).

**FIGURE 4:**
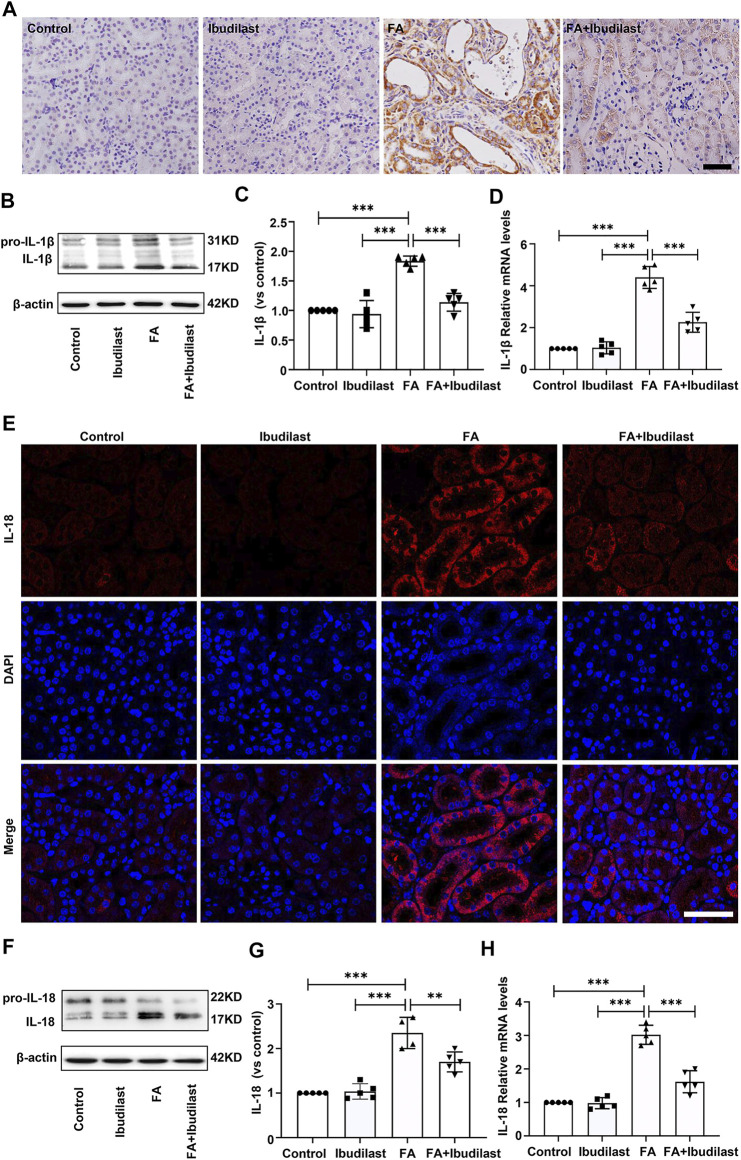
Ibudilast administration decreased the expression of pyroptotic markers (IL-1β and IL-18) in mice with FA-induced AKI. **(A)** Immunohistochemical labeling for IL-1β, bar = 50 μm. **(B)** Western blot showing the levels of pro-IL-1β and IL-1β. **(C)** Semiquantitative measurements of IL-1β. **(D)** Relative mRNA expression of IL-1β. (E) Immunofluorescence staining for IL-18, bar = 50 μm. **(F)** Western blot of pro–IL-18 and IL-18. **(G)** Semiquantitative measurements of IL-18. **(H)** Relative mRNA expression of IL-18. Data are presented as the mean ± SEM, *n* = 5. *p < 0.05, **p < 0.01, and ***p < 0.001.

Next, we investigated the levels of the pyroptosis-related markers, IL-1β and IL-18, which mediate the proteolytic maturation of pro-IL-1β and pro-IL-18 by activated caspase-1. IHC staining of kidney sections was used to evaluate the changes in the expression of the pro-inflammatory factor IL-1β, revealing significantly increased levels of IL-1β after FA injection, while ibudilast administration markedly downregulated the numbers of IL-1β–positive cells in FA-injured kidneys (Figure 4A). These findings were further verified by Western blots that showed approximately 1.8-fold higher levels of active IL-1β in FA-injected mice, but these changes were reduced by approximately 38% upon ibudilast administration (Figures 4B,C). Moreover, the FA injection significantly stimulated the expression of the IL-1β mRNA relative to the control kidney (approximately 4.4-fold), and ibudilast administration attenuated the increase by 48.6% (Figure 4D). Furthermore, immunofluorescence labeling showed that the FA injection increased the number of IL-18–positive tubular cells in the FA group, but this change was alleviated in the FA + ibudilast group (Figure 4E). Likewise, Western blot assays indicated that the FA injection promoted conversion of pro–IL-18 to the active form, with an increase in its level by approximately 2.3-fold: a change that was attenuated by 27.7% upon ibudilast administration (Figures 4F,G). Moreover, we determined the expression of the IL-18 mRNA with qRT-PCR and found that ibudilast administration decreased the FA-induced increase in the level of the IL-18 mRNA by approximately 46%, as illustrated in Figure 4H.

**FIGURE 5:**
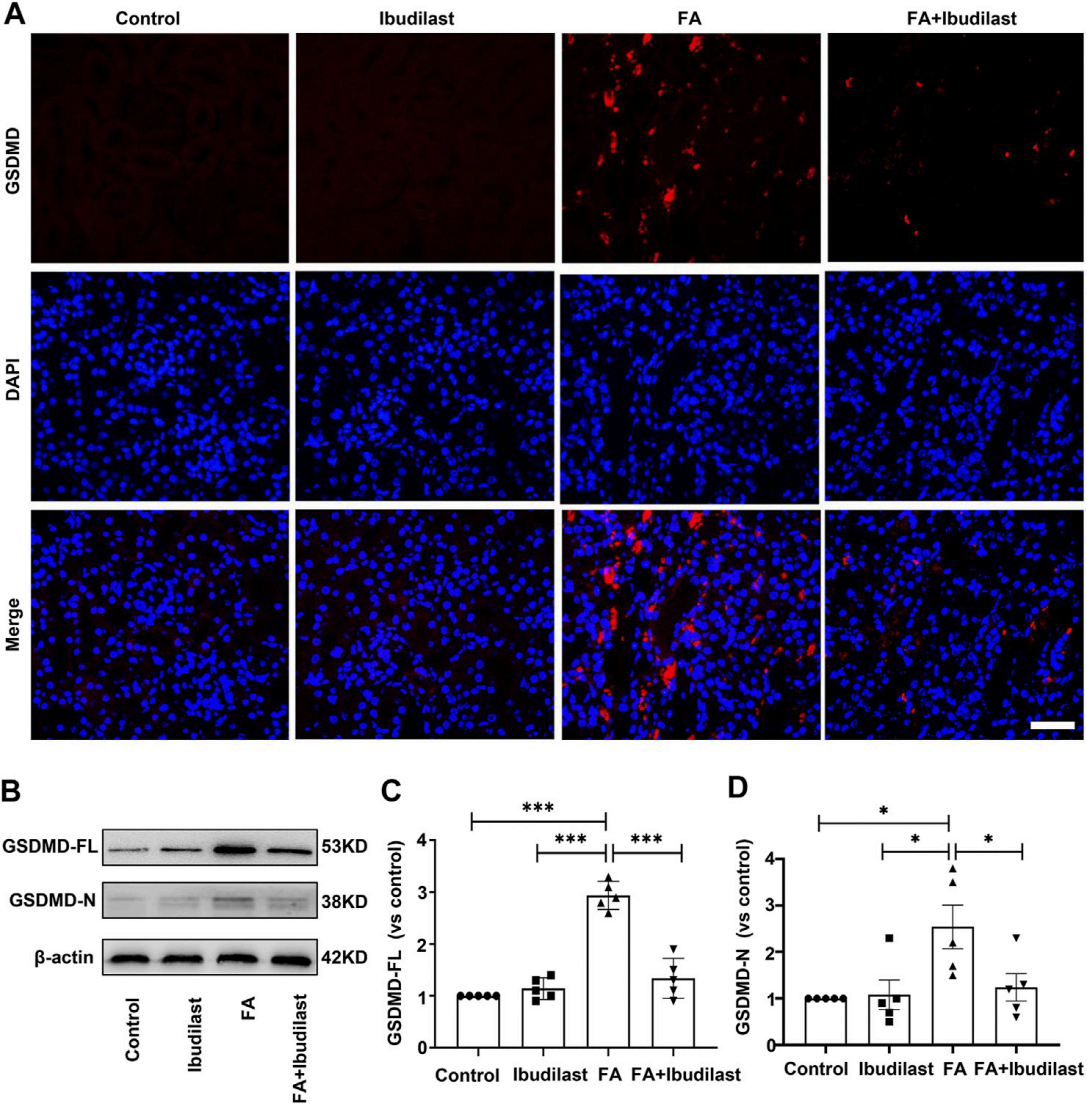
Ibudilast administration decreased the level of the pyroptosis-related molecule GSDMD in mice with FA-induced AKI. **(A)** Immunofluorescence labeling of GSDMD, bar = 50 μm. **(B)** Western blot showing levels of the full-length GSDMD (GSDMD-FL) and active forms of GSDMD (GSDMD-N). **(C)** Semiquantitative measurements of GSDMD-FL. **(D)** Semiquantitative measurements of GSDMD-N. Data are shown as the mean ± SEM, *n* = 5. *p < 0.05, **p < 0.01, and ***p < 0.001.

Furthermore, GSDMD is cleaved by activated caspase-1 and then forms pores that promote cell pyroptosis (Semino et al., 2018). As depicted in Figure 5A, immunofluorescence labeling showed a substantial increase in the expression of GSDMD in FA-injected mice, which was drastically reversed by ibudilast administration. Consistent with the findings described above, Western blot analysis showed that both GSDMS-FL and GSDMD-N fragments were increased (approximately 2.9-fold and 2.5-fold, respectively) in the kidneys of FA-injected mice, while these effects were inhibited by 54.4 and 51.2%, respectively, upon ibudilast administration (Figures 5B–D).

**FIGURE 6:**
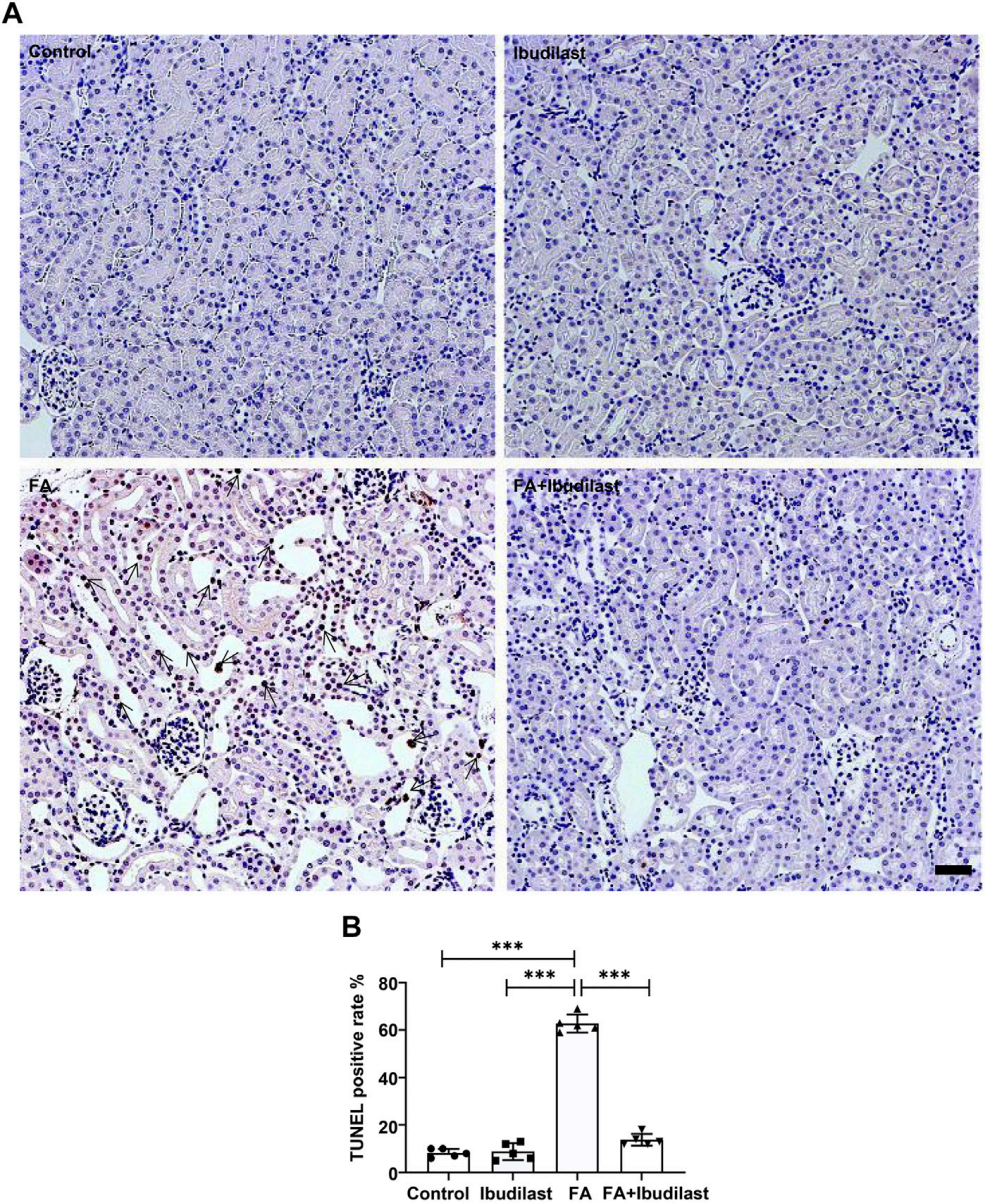
Ibudilast administration decreased pyroptosis-induced cell death in mice with FA-induced AKI. **(A)** Representative results of the TUNEL assay in kidney slices. Arrowheads indicate TUNEL-positive tubular cells, bar = 50 μm. **(B)** Quantification of TUNEL-positive cells. Data are presented as the mean ± SEM, *n* = 5. *p < 0.05, **p < 0.01, and ***p < 0.001.

In addition, we performed a TUNEL assay to examine DNA fragmentation and determine whether caspase-1–dependent cell death occurred in our study. The FA group showed numerous TUNEL-positive tubular epithelial cells (62.8 ± 3.77%), while the FA + ibudilast group displayed a dramatically reduced number of TUNEL-positive cells (13.8 ± 2.49%), as shown in Figures 6A,B.

Collectively, these results indicated that ibudilast administration was capable of reducing tubular epithelial cell pyroptosis induced by FA injection *in vivo*.

### Ibudilast Increased the Level of the Anti-Inflammatory Factor IL-10 and Inhibited the Production of the Pro-inflammatory Cytokine TNF-α and Macrophage Infiltration in Mice With Folic Acid–Induced Acute Kidney Injury

**FIGURE 7:**
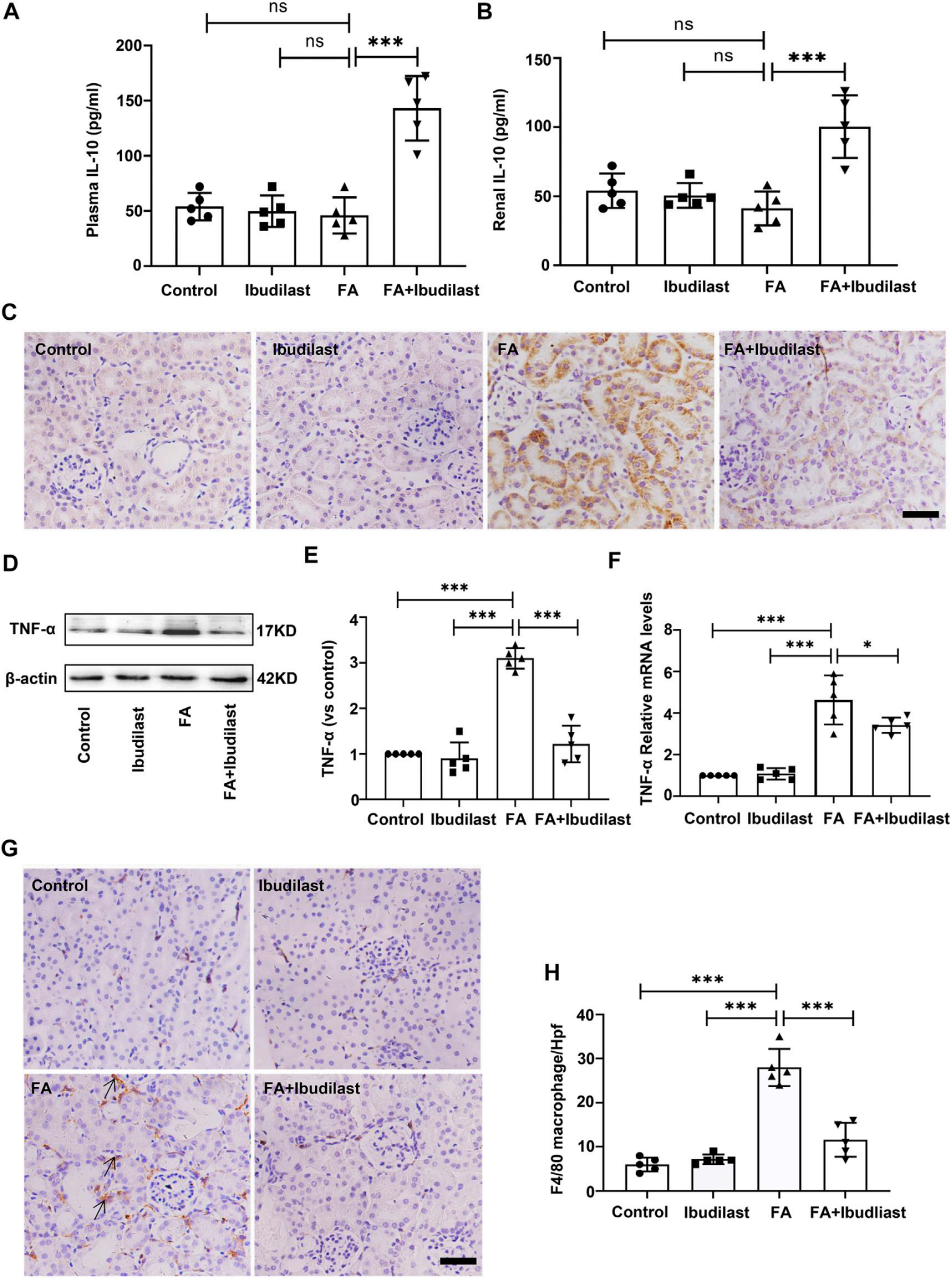
Ibudilast administration increased the levels of IL-10 and decreased TNF-α secretion and macrophage infiltration in mice with FA-induced AKI. **(A)** The level of IL-10 in serum. **(B)** The level of IL-10 in kidney tissue homogenates. **(C)** Immunohistochemical labeling of TNF-α, bar = 50 μm. **(D)** Western blot for TNF-α. **(E)** Semiquantitative measurements of TNF-α. **(F)** Relative mRNA expression of TNF-α. **(G)** Immunohistochemical labeling for macrophages. **(H)** Absolute count of macrophages per high-power field (Hpf). Data are presented as the mean ± SEM, *n* = 5. *p < 0.05, **p < 0.01, and ***p < 0.001.

We performed experiments to assess whether the inhibition of pyroptosis in ibudilast-treated mice after FA injection was associated with less inflammation. The levels of the anti-inflammatory cytokine IL-10 were detected using an ELISA. As shown in Figures 7A,B, the levels of IL-10 were not significantly decreased in both serum and renal tissue of FA-injected mice (46.0 ± 16.39 pg/ml and 41.2 ± 12.28 pg/ml, respectively), while ibudilast treatment reversed their expressions (143.2 ± 29.29 pg/ml and 100.4 ± 22.62 pg/ml, respectively). Moreover, the level of pro-inflammatory cytokine TNF-α was examined in the kidney using immunohistochemical labeling. As depicted in Figure 7C, TNF-α–positive cells were mainly localized in tubular epithelial cells, and their numbers were dramatically increased in the FA-injected mice compared with non–FA-injected mice, while this effect was abrogated by ibudilast administration. Consistent with these observations, Western blot and qRT-PCR assays showed that ibudilast administration significantly attenuated FA-induced increase in the expression of the TNF-α protein and mRNA by approximately 60.6 and 26.3%, respectively (Figures 7D–F). Additionally, immunohistochemical staining for macrophages showed that F4/80-positive cells were distributed in the interstitial compartment and significantly increased in number in the FA group (28 ± 4.18/HP), while the FA + ibudilast group exhibited fewer F4/80-positive cells in this space (11.6 ± 3.85/HP; Figures 7G,H).

### Effects of Ibudilast on the Toll-Like Receptor 4 Mediated NF-κB and Mitogen-Activated Protein Kinase Signaling Pathways in Mice With Folic Acid–Induced Acute Kidney Injury

**FIGURE 8:**
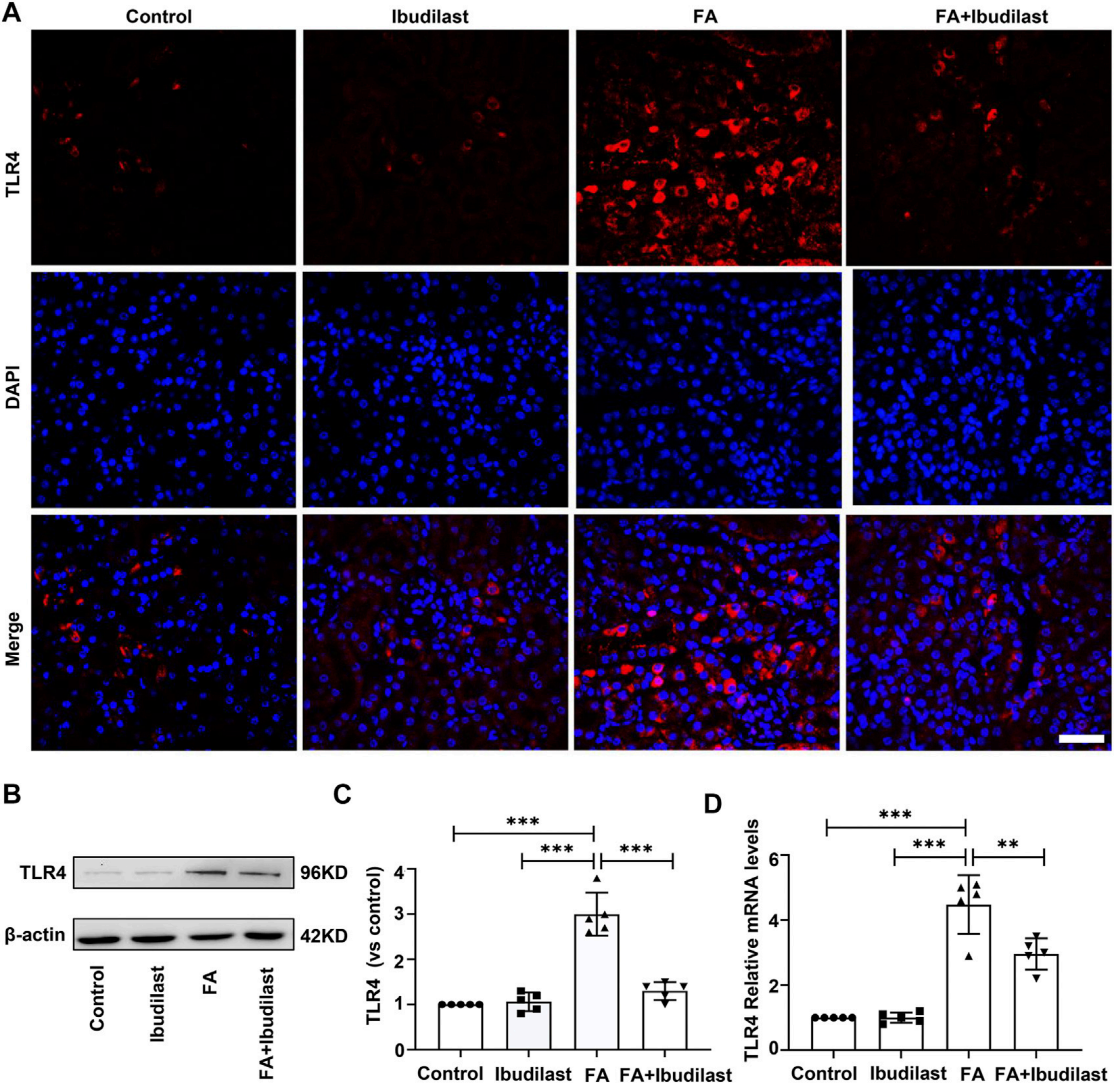
Ibudilast administration inhibited the expression of TLR4 in mice with FA-induced AKI. **(A)** Immunofluorescence labeling of TLR4, bar = 50 μm. **(B)** Western blot showing TLR4 levels. **(C)** Semiquantitative measurements of TLR4. **(D)** Relative mRNA expression of TLR4. Data are shown as the mean ± SEM, *n* = 5. *p < 0.05, **p < 0.01, and ***p < 0.001.

We first investigated the effect of ibudilast on the expression of TLR4 in mice with FA-induced acute tubular injury to explore the mechanism underlying the anti-pyroptotic effect of ibudilast. The results of immunofluorescence staining revealed that the FA injection induced a significant upregulation of TLR4, while this effect was abolished by ibudilast administration (Figure 8A). Consistently, the Western blot analysis showed that ibudilast administration inhibited the FA-induced increase in TLR4 expression by 56.7%, as shown in Figures 8B,C. These results were consistent with our qRT-PCR data shown in Figure 8D, substantiating the finding that ibudilast treatment reduced the FA-induced expression of the TLR4 mRNA induced by approximately 33.9%.

**FIGURE 9:**
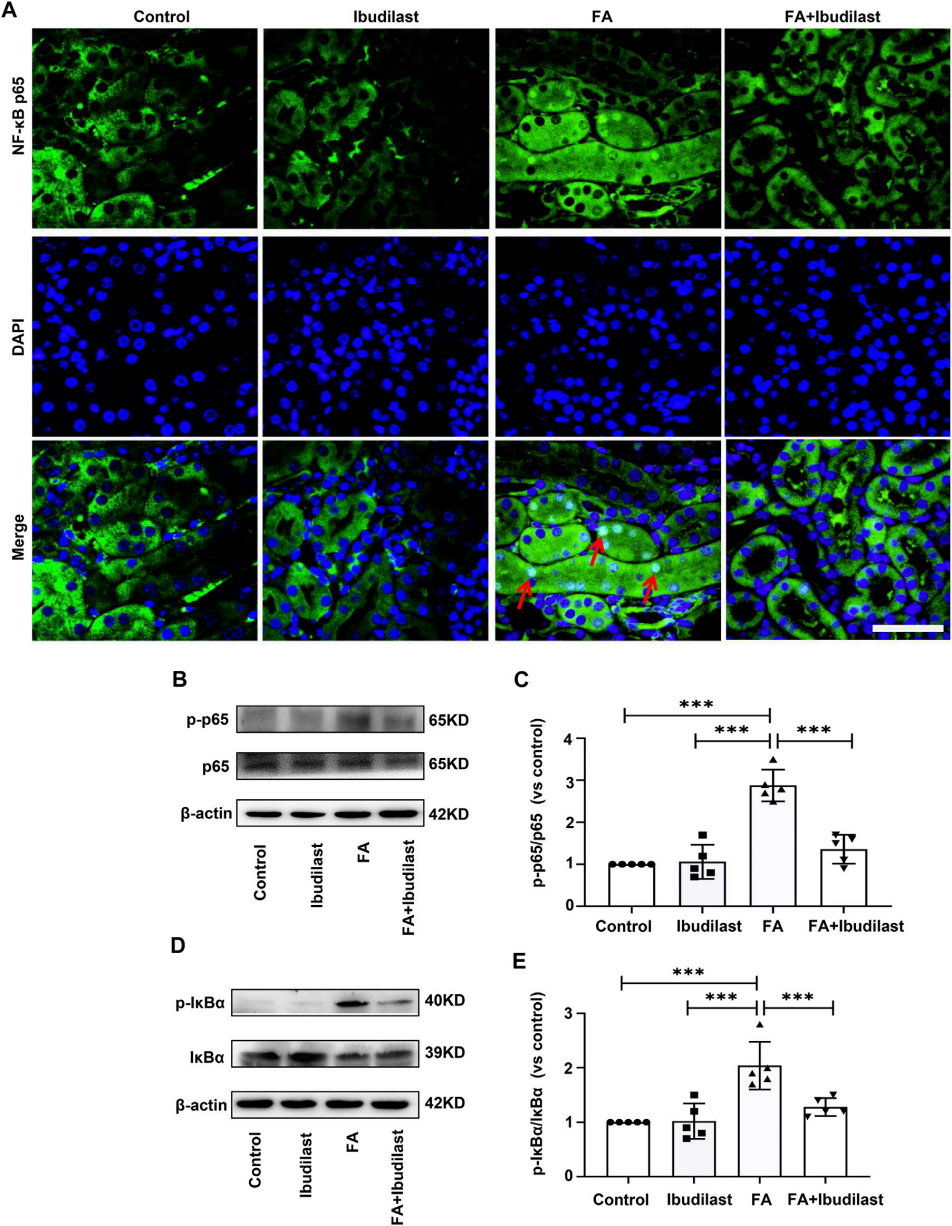
Ibudilast administration downregulated the NF-κB signaling pathway in mice with FA-induced AKI. **(A)** Immunofluorescence staining showing NF-κB p65 nuclear translocation (red arrowheads), bar = 50 μm. **(B)** Western blot showing p-p65 and p65 levels. **(C)** Semiquantitative measurements of the ratio of p-p65 to p65. **(D)** Western blot showing *p*-IκBα and IκBα levels. **(E)** Semiquantitative measurements of the ratio of *p*-IκBα to IκBα. Data are presented as the mean ± SEM, *n* = 5. *p < 0.05, **p < 0.01, and ***p < 0.001.

Furthermore, we examined the effect of ibudilast administration on the classic TLR4/NF-κB inflammatory signaling pathway in FA-induced acute tubular injury. Immunofluorescence staining revealed that NF-κB p65 was mainly located in the cytoplasm of tubular epithelial cells in non–FA-injected mice, but extensive nuclear translocation of NF-κB p65 was observed after FA injection. However, ibudilast administration significantly reduced the nuclear accumulation of NF-κB p65 (Figure 9A). Likewise, Western blot analysis indicated that FA injection induced an increase in the level of p-p65 by approximately 2.9-fold, while this effect was attenuated by approximately 52.8% upon ibudilast administration (Figures 9B,C). Furthermore, ibudilast inhibited FA-induced phosphorylation of IκBα by approximately 37.3%, as shown in Figures 9D,E.

**FIGURE 10:**
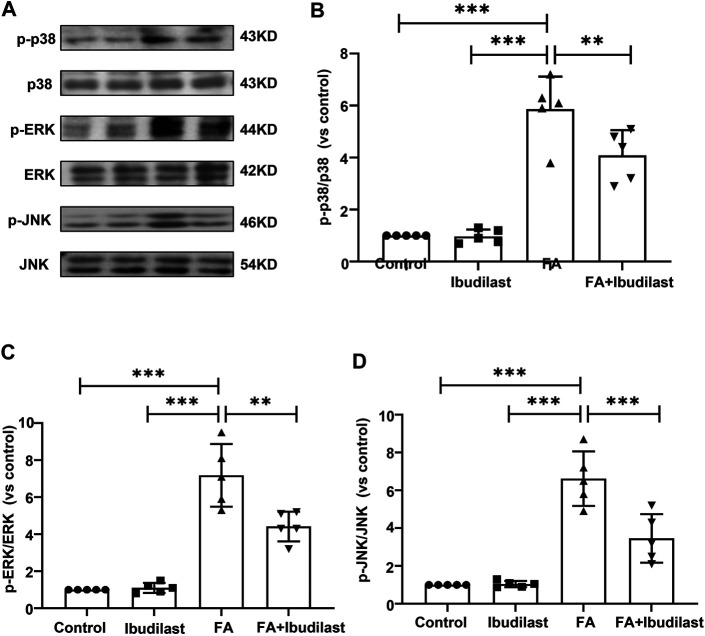
Ibudilast administration inhibited the MAPK signaling pathway in mice with FA-induced AKI. **(A)** Western blots showing *p*-p38, p38, *p*-ERK, ERK, *p*-JNK, and JNK levels. **(B)** Semiquantitative measurements of p-p38 to p38. **(C)** Semiquantitative measurements of *p*-ERK to ERK. **(D)** Semiquantitative measurements of *p*-JNK to JNK. Data are shown as the mean ± SEM, *n* = 5. *p < 0.05, **p < 0.01, and ***p < 0.001.

In addition, increasing evidence has revealed that renal inflammation is closely related to MAPK signaling pathway activation. We further characterized the phosphorylation of p38, ERK, and JNK, the three major subfamilies of kinases involved in MAPK signaling, using Western blotting (Figure 10A). The phosphorylation of p38, ERK, and JNK was significantly increased after the FA injection by approximately 5-fold, 7-fold, and 6-fold, respectively, while the ibudilast treatment reduced their levels by approximately 30.4, 38.4, and 47.7%, respectively.

## Discussion

Kidney injuries induced by FA are mainly due to folic crystal obstruction and oxidative stress, which lead to tubular injury, a common pathological process of AKI (Agarwal et al., 2016). Based on accumulating evidence, the inflammatory response exerts a key pathogenic effect on the progression of FA-induced AKI (Aparicio-Trejo et al., 2019), the features of which are similar to those of clinical AKI (Gupta et al., 2012; Li X. et al., 2020). A previous study showed that ibudilast, an anti-inflammatory agent, has been widely used to treat asthma (Hama et al., 2012). In addition, ibudilast has been used to alleviate morphine-induced CNS inflammation through TLR4 signaling inhibition (Zhaleh et al., 2014). In this study, we showed that ibudilast, a TLR4 blocker, improved the damaged renal function in mice with FA-induced AKI by blocking TLR4 activation, serving as a therapeutic option that induces anti-inflammatory mechanisms. The inflammation induced by an overdose FA injection may be involved in the release of DAMPs from damaged or necrotic tubular cells, which directly or indirectly activate TLR4 signaling. Our results were consistent with those of a previous study showing that TLR4 is activated by endogenous proteins released from damaged tissue and participates in mediating renal injury following ischemia and reperfusion (I/R), which is alleviated in TLR4 mutant mice (Souza et al., 2015).

Pyroptosis is recognized as a unique inflammatory form of regulatory cell death that is mainly mediated by NLRP3 inflammasome activation. Pyroptosis not only contributes to infectious diseases (Zhang et al., 2018) but also participates in sterile inflammatory diseases, including gout, diabetes, asbestosis, and metabolic syndrome, which might further induce kidney injury (Gaidt et al., 2016; Chen et al., 2018; Hughes and O'Neill 2018; Wang et al., 2019; Mouasni et al., 2019). Increasing evidence has shown that programmed cell death, including pyroptosis, rather than apoptosis, plays an essential role in the pathological progression of various types of AKI (Hughes and O’Neill 2018; Wang Y. et al., 2019; Mouasni et al., 2019). Activation of the NLRP3 inflammasome was reported to be the key mediator of I/R-induced renal injuries (Yang et al., 2014; Wang L. et al., 2019). Consistently, in the present study, the expression of NLRP3 was upregulated by FA injection, indicating that pyroptosis initiated by the activation of the NLRP3 inflammasome may be the potential mechanism underlying FA-induced tubular injury. Ibudilast administration significantly reduced the level of the NLRP3 inflammasome. This finding was consistent with those of previous studies showing that the inhibition of NLRP3 inflammasome activation alleviates tubular injury in cisplatin-injected mice (Li S. et al., 2019) and that NLRP3 knockout mice exhibit attenuated UUO-induced kidney injury (Vilaysane et al., 2010).

In addition, the NLRP3 inflammasome has been implicated in the activation of caspase-1, which further promotes the cleavage of inflammatory precursors into active forms, such as IL-1β and IL-18, which marks pyroptosis progression (Dai et al., 2019). As shown in the present study, the pyroptotic markers caspase-1, IL-18, and IL-1β were mainly expressed in the cytoplasm of tubular epithelial cells, and their levels were obviously increased by the FA injection, but the expression of these pyroptotic markers was inhibited by ibudilast administration. This finding was consistent with that of a previous study showing that the inhibition of caspase-1 alleviates tubular damage by attenuating the transition of pro-inflammatory precursors into active forms in mice with IRI-induced AKI [1]. Numerous investigators have documented that tubular cell pyroptosis is reduced by caspase-1 inhibition (Wang L. et al., 2019). Furthermore, pyroptosis is a form of lytic cell death that occurs through the formation of plasma membrane pores, and GSDMD, a caspase substrate, is required for pyroptotic pore formation and inflammatory factor secretion (Semino et al., 2018; Sborgi et al., 2016). Miao et al. found that GSDMD mediates tubular epithelial cell pyroptosis and facilitates the release of IL-18 after cisplatin administration (Miao et al., 2019). In contrast, GSDMD knockout mice showed an amelioration of cisplatin-induced AKI (Li Y. et al., 2020). The present study coincidently revealed that more pores were formed on the plasma membrane of tubular cells after the FA injection, which was effectively inhibited by ibudilast administration.

TUNEL staining, which indicates DNA breakage in cells, is frequently used as an apoptotic and pyroptotic marker (Luo et al., 2014; Ye Z. et al., 2019). We detected more TUNEL-positive tubular epithelial cells in the FA-injected mice, which was consistent with increases in NLRP3, caspase-1, IL-1β, and GSDMD cleavage. Ibudilast administration obviously decreased the number of TUNEL-positive tubular epithelial cells. Collectively, our findings indicated that ibudilast administration suppressed tubular cell pyroptosis induced by the FA injection.

Pyroptosis is even more critical for kidney injury than apoptosis due to its perpetual pro-inflammatory cascade in the tissue (Cheng et al., 2019). During pyroptosis, pro-inflammatory cytokines and chemotactic factors are released to recruit additional immune cells, producing more inflammatory cytokines and aggravating tubular epithelial cell damage (Song et al., 2019; Xu et al., 2020). In the present study, we confirmed that the FA injection significantly promoted the release of TNF-α and macrophage infiltration in mouse kidneys: changes that were effectively reduced by ibudilast administration. Moreover, IL-10 is a pluripotent cytokine involved in the suppression of inflammation, and targeted therapy to increase its level could alleviate renal damage (Soranno et al., 2016). It has been reported that renal ischemia-reperfusion injury can induce an increase in IL-10 (Patil et al., 2016), while cisplatin administration prominently reduced its expression (Honarpisheh et al., 2018). In our study, the level of IL-10 was slightly diminished after FA injection, but ibudilast treatment significantly increased its level both in serum and renal tissue. It was consistent with the study that FA injection caused a moderate reduction in IL-10, which may correlate with severe oxidative stress injury that impaired its upregulation (Honarpisheh et al., 2018).

Moreover, TLR-4/NF-κB signaling has a central role in regulating the release of pro-inflammatory cytokines, and therapeutic agents targeting this signaling pathway significantly reduce inflammation (Ahmad et al., 2015). NF-κB is an inducible transcriptional element that is located in the cytoplasm when inactivated (Nadeem et al., 2017). Iκ-Bα is a critical regulator of NF-κB, and NF-κB translocates into the nucleus following IκBα phosphorylation (Courtois and Gilmore 2006). The inhibition of NF-κB reduces the expression of its downstream effector IL-8 and suppresses caspase-dependent apoptosis, which protects human medulloblastoma cells from oxidative stress injury (Ashour et al., 2016). Furthermore, accumulating evidence indicates that the activation of the NLRP3 inflammasome, which is involved in pyroptosis, is also closely associated with the NF-κB signaling pathway (Lei et al., 2018; Zhang et al., 2019). Two signals are required for NLRP3 inflammasome activation: the first priming signal results from NF-κB pathway–mediated upregulation of NLRP3 expression, and the second signal is transduced by PAMPs/DAMPs, which activate the functional NLRP3 inflammasome following the activation of caspase-1 to process and release inflammatory cytokines (Lei et al., 2018; Zhang et al., 2019). A recent study showed that emodin alleviates I/R-induced cardiomyocyte injury by inhibiting pyroptosis via the suppression of NF-κB–mediated NLRP3 inflammasome activation (Ye B. et al., 2019). Another study reported that andrographolide ameliorates intracerebral hemorrhage by blocking the NF-κB/NLRP3 inflammasome pathway (Wang et al., 2018). In addition, ibudilast has been shown to alleviate Alzheimer’s disease by reducing the production of inflammatory factors via the downregulation of NF-κB (Wang et al., 2014; Schwenkgrub et al., 2017). In conjunction with previous findings, the present study indicated that ibudilast administration effectively blocked the pro-inflammatory response by downregulating the TLR4/NF-κB-mediated pathway and inhibiting NLRP3 inflammasome activation in an FA-induced AKI model.

Moreover, emerging data suggest that the MAPK pathway plays an important role in causing renal inflammation, which might be activated by hyperactivated TLR4 (Gao et al., 2020). In addition, an overdose FA injection induced the activation of the MAPK pathway, which is the main target involved in the protective effect on FA-induced AKI (Jiang et al., 2016). A recent study reported that NCS613, another potent PDE4 inhibitor, was a negative modulator of p38 phosphorylation that exerts anti-inflammatory effects on TNF-α–treated lung epithelial cells by inhibiting the activation of the MAPK signaling pathway (Yougbare et al., 2020). Moreover, some studies have suggested that the MAPK signaling pathway mainly contributes to cell pyroptosis, which further drives massive inflammation (Yao and Sun 2019). We detected the levels of phosphorylated p38, ERK, and JNK to determine whether ibudilast reduced renal inflammation by inhibiting the MAPK signaling pathway. The phosphorylation of these kinases was significantly increased by the FA injection, while ibudilast administration markedly reduced their phosphorylation levels. Based on our results, ibudilast administration inhibited FA-induced JNK, ERK, and p38 phosphorylation in the kidney, suggesting that ibudilast might also reduce pyroptosis and inflammation by inhibiting the MAPK pathway.

However, this study has some limitations. For example, our experiment mainly focuses on *in vivo* experiments, and further cell experimentation may be needed to explore the underlying protective mechanisms of ibudilast in the presence of the related inhibitors, which may help to clarify the most important targeted mitigation of FA-induced AKI by ibudilast. Moreover, it may be preferable to provide the insights into the cross talk between the NF-kB and MAPK signaling pathways involved in regulating the anti-inflammatory response of ibudilast in the near future.

In conclusion, our study provided evidence that an overdose FA injection in mice induced tubular pyroptosis and inflammation, possibly through TLR4-mediated NF-κB and MAPK signaling pathways, which were suppressed by ibudilast administration, providing a novel insight into the overall protective effect of ibudilast on AKI.

## Data Availability

The original contributions presented in the study are included in the article/Supplementary Material; further inquiries can be directed to the corresponding author.
